# Informational continuity of medication management in transitions of care: Qualitative interviews with stakeholders from the HYPERION-TransCare study

**DOI:** 10.1371/journal.pone.0300047

**Published:** 2024-04-04

**Authors:** Truc Sophia Dinh, Maria Hanf, Astrid-Alexandra Klein, Maria-Sophie Brueckle, Lisa Rietschel, Jenny Petermann, Franziska Brosse, Sylvia Schulz-Rothe, Sophia Klasing, Christiane Muth, Hanna Seidling, Jennifer Engler, Karola Mergenthal, Karen Voigt, Marjan van den Akker

**Affiliations:** 1 Institute of General Practice, Goethe-University Frankfurt, Frankfurt am Main, Germany; 2 Department of General Practice/Medical Clinic III, Faculty of Medicine Carl Gustav Carus, TU Dresden, Dresden, Germany; 3 Department of Clinical Pharmacology and Pharmacoepidemiology, Heidelberg University Hospital, Heidelberg, Germany; 4 Cooperation Unit Clinical Pharmacy, Heidelberg University, Heidelberg, Germany; 5 Department of General Practice and Family Medicine, Medical School Westphalia, Bielefeld University, Bielefeld, Germany; 6 Department of Family Medicine, Care and Public Health Research Institute, Maastricht University, Maastricht, The Netherlands; 7 Department of Public Health and Primary Care, Academic Centre of General Practice, KU Leuven, Leuven, Belgium; King Saud University Medical City, SAUDI ARABIA

## Abstract

**Background:**

The transition of patients between inpatient and outpatient care can lead to adverse events and medication-related problems due to medication and communication errors, such as medication discontinuation, the frequency of (re-)hospitalizations, and increased morbidity and mortality. Older patients with multimorbidity and polypharmacy are particularly at high risk during transitions of care. Previous research highlighted the need for interventions to improve transitions of care in order to support information continuity, coordination, and communication. The HYPERION-TransCare project aims to improve the continuity of medication management for older patients during transitions of care.

**Methods and findings:**

Using a qualitative design, 32 expert interviews were conducted to explore the perspectives of key stakeholders, which included healthcare professionals, patients and one informal caregiver, on transitions of care. Interviews were conducted between October 2020 and January 2021, transcribed verbatim and analyzed using content analysis. We narratively summarized four main topics (stakeholders’ tasks, challenges, ideas for solutions and best practice examples, and patient-related factors) and mapped them in a patient journey map. Lacking or incomplete information on patients’ medication and health conditions, inappropriate communication and collaboration between healthcare providers within and across settings, and insufficient digital support limit the continuity of medication management.

**Conclusions:**

The study confirms that medication management during transitions of care is a complex process that can be compromised by a variety of factors. Legal requirements and standardized processes are urgently needed to ensure adequate exchange of information and organization of medication management before, during and after hospital admissions. Despite the numerous barriers identified, the findings indicate that involved healthcare professionals from both the inpatient and outpatient care settings have a common understanding.

## Introduction

The transition of patients at the interface between inpatient and outpatient care bears the risk of discontinuation in patients’ medication, which can lead to negative patient-relevant outcomes such as increased re-hospitalization and higher all-cause mortality [1–4]. Reasons for discontinuation include medication and communication errors (e.g., failure to communicate changes in medication, incomplete medical records [5, 6] or incorrectly reported medications [7]) that can result in medication-related problems [2, 8]. These refer, for example, to drug selection, route of administration or dose selection [9]. In the context of transitions of care, these problems can occur before, during or after a hospital stay [10, 11]. Up to 20% of hospitalizations, including re-hospitalizations, can be attributed to medication-related problems [12]. The prevalence of medication-related problems after discharge ranges from 14 to 49% [13]. The study by El Morabet et al. [12] examines various types of re-hospitalization related to drugs, encompassing due to: drug-related problems (DRPs), adverse drug events (ADEs), adverse drug reactions (ADRs), and medication errors (MEs). Approximately half of the MEs occurring in hospitals are estimated to take place during admission or discharge from hospital [14], such as when compiling the patient’s medication history or during prescription [15]. In an outpatient setting, about 10% of the Slovenian population faces ADEs due to clinically relevant potential drug-drug interactions (DDIs) [16], with a higher proportion among older people. Additionally, a review showed that in 4.8% of admissions, DDIs were the reported cause [17]. In four German emergency departments ADRs were a relevant reason in 6.5% of emergency admissions, often leading to inpatient admission [18].

In transitions of care, particularly older patients with multimorbidity and polypharmacy constitute a population at high risk. The care journeys for these patients are complex and involve healthcare professionals (HCPs) from different care settings with multiple tasks, interfering in own working procedures [19–24]. Pharmacists can play a key role in improving the quality of medication histories and updating patient records [25, 26]. To ensure continuity of care, patients’ care and health-related information must not only be coordinated between these HCPs [20, 22, 27], but also between HCPs and patients and their caregivers (CG) [1, 2, 28, 29]. Communication between inpatient and outpatient physicians is infrequent [30] and the lack of standardized processes for communication in transitions of care poses a significant problem [21, 30].

According to statutory discharge management, German hospitals are required by law to prepare a discharge in order to ensure continuous patient care after discharge. However, a scoping review with a focus on older, impaired patients identified numerous gaps in discharge management and hospital care in general for this patient group [31].

Previous research has therefore underscored the need for interventions to improve transitions of care in order to support information continuity, coordination and communication between different care settings [19, 21, 30]. This includes, for example, establishing interconnected electronic information support systems and developing navigator roles [32]. Furthermore, an increase of informational continuity can help to improve quality of care [28] and to empower patients to self-manage their own care [8, 33]. A UK-based study [34, 35] testing an intervention to improve medication management during transition of care in patients with heart failure showed that discharged patients wished to have knowledge regarding the effects and potential side effects of their medications. The study also found that the intervention did enhance knowledge among patients and relatives and improve communication among professionals, patients, and their families.

The HYPERION-TransCare project was created for this same reason, aimed at the development of a complex intervention to improve informational continuity of medication management for older patients in transitions of care. Key stakeholders were therefore interviewed in a first step in order to understand their views and needs. The results of these interviews are described in this paper.

## Materials and methods

### HYPERION-TransCare project

HYPERION-TransCare (Heading to continuitY of Prescribing in EldeRly with multImOrbidity iN Transitional Care) is a project conducted under the umbrella of the practice-based research network (PBRN) *SaxoForN* that is being established between the two German states Saxony and Hesse [36–38]. In the context of the study, the term ‘transitions of care’ refers to the transition of patient care from the outpatient care setting—meaning care provided outside the hospital setting—to the inpatient care setting (hospital referral and hospital admission) and vice versa (hospital discharge and follow-up).

The research project comprises two sub-studies. Sub-study 1 concerns the development of a complex intervention that improves continuity of medication management in transitions of care. An ethical review and approval were waived for this study by the Ethics Committee of Goethe University Frankfurt on September 4, 2020 due to the nature of this study (qualitative stakeholder analysis based on expert interviews). Participants provided written informed consent before participating in the study. The rationale of sub-study 1 was previously published in a study protocol [39]. Sub-study 1 is divided into two parts. The first part reported here is aimed at exploring, understanding and describing the care setting and stakeholder-specific perspectives, experiences and needs regarding continuity of medication management during care transitions, using qualitative expert interviews. In the second part, the qualitative expert interview results were further analyzed in co-design workshops along with representatives of these different stakeholder groups to come up with a new intervention to improve continuity of medication management in transitions of care [39, 40]. The recruitment of stakeholders and the results of the workshops are published elsewhere [40]. Based on the results of the co-design workshops, the intervention is currently being tested in an interventional study.

### Study design

Qualitative expert interviews were used to explore participants’ perspectives on 1) tasks, 2) challenges, 3) solution ideas and best practice examples, and 4) patient-related factors with regard to transitions of care [41, 42]. In the context of our study, experts are deemed as having specialized knowledge and experience related to older patients’ medication at the interface of inpatient and outpatient care [43, 44]. This paper adheres the Consolidated Criteria for Reporting Qualitative Research (COREQ, S1 Checklist) [45].

### Participant selection and recruitment

We aimed to include a heterogeneous sample of HCPs, patients and informal caregivers. We therefore used purposive sampling techniques [46] to recruit a wide range of professions in the care setting of interest in terms of sex and rural/urban area dwelling. The purposive sampling approach allowed the study team to select individuals or groups who were particularly familiar with the phenomenon of interest, taking into account criteria such as participant knowledge, experience, availability, and willingness to participate [46, 47]. We contacted participants via phone or e-mail. More details were published separately in the study protocol [38]. To be eligible for the study, HCPs were required to be involved in patients’ medication in transitions of care. Therefore, we aimed to recruit general practitioners (GPs), healthcare assistants (HCAs) and ambulatory care nurses (ACNs) all from the outpatient care setting. To cover the inpatient care setting in the study, the plan was to invite inpatient physicians (IPs), inpatient nurses (INs) and hospital pharmacists (HPs) to participate. We also aimed to include the perspective of clinical information scientists (CISs) [39]. Patients were eligible as experts if they had multimorbidity (co-existence of two or more chronic conditions [48]) and polypharmacy (≥ 5 medications at the same time [49]), and if they had been hospitalized in the last 3 months. Study eligibility for informal caregivers required them to be caring for a relative who met the aforementioned inclusion criteria. GPs and healthcare assistants were to be recruit via the existing trans-regional PBRN *SaxoForN* [50], enabling us to draw on existing collaborations and contacts with experienced HCPs in teaching, education and research fields. Patients and informal caregivers were to be recruited via general practices in our PBRN and through announcements in local newspapers in Hesse and Saxony. The selection of patients and informal caregivers was based on the inclusion criteria focusing on their personal experiences with multimorbidity and polypharmacy. We planned to recruit hospital pharmacists from Saxony as a best practice. Recruitment of all other participants was planned to be undertaken through the meticulously cultivated network of institutional and personal contacts within the PBRNs. Potential participants received written information about the study and data protection. If they were interested in study participation, one member of the study team (FB, M-SB, TSD, A-AK, JP, LR, SSR) provided detailed information about the study and made an appointment for an interview. Once accepted, participants received an informed consent form and a short questionnaire, and were asked to fill out and return both prior to the interviews. They were offered €50 for participation.

### Data collection

#### Expert interviews

The research team (FB, M-SB, TSD, JE, A-AK, KM, JP, LR, MvdA, KV) developed nine stakeholder-specific, semi-structured interview guides based on the “Manual for Conducting Qualitative Research” by Helfferich [51], and covering collecting, checking, sorting and subsuming research questions. This yielded interview guides for HCPs focused on relevant stakeholders and their tasks, challenges, ideas for solutions and best practice examples regarding medication management in transitions of care. Clinical information scientists were asked to provide their view on the current situation concerning the exchange of information between care settings including challenges and ideas for solutions. The interview guides for patients and caregivers concentrated on their most recent hospital admission including their challenges and ideas for solutions. Patients were interviewed as experts on their own behalf and based on their own experiences. Examples of an interview guide for each inpatient and outpatient HCP group and patient stakeholder group can be found in S1–S3 Files.

Prior to conducting the interviews, one interview guide (GP guide) serving as an example was discussed in depth in an interdisciplinary qualitative research group at the Institute of General Practice in Frankfurt. Five researchers (M-SB, TSD, A-AK, LR, KV) then pre-tested all interview guides in pilot interviews and adapted them accordingly. Between October 2020 and January 2021, the same researchers conducted the individual expert interviews by telephone, using the web-hosted service GoToMeeting [52]. Four of these researchers were experienced in conducting qualitative interviews, and the fifth was carefully instructed by the experienced team members. The interviews were audio-recorded with the consent of participants, and transcribed verbatim by a professional transcription office and experienced staff of the Department of General Practice / Medical Clinic III, Faculty of Medicine Carl Gustav Carus, TU Dresden, Germany and the Institute of General Practice, Goethe-University Frankfurt am Main, Germany. To ensure data protection, transcripts were anonymized and pseudonyms were created for the presentation of results (“GP” for general practitioner, “HCA” for healthcare assistant, “ACN” for ambulatory care nurse, “IP” for inpatient physician, “IN” for inpatient nurse, “HP” for hospital pharmacist, “CG” for caregiver and “CIS” for clinical information scientist, plus an individual number, and “f” for female or “m” for male). Transcripts were not returned to participants for comments and/or corrections. Field notes were taken after the interviews.

#### Questionnaires

Interview participants received stakeholder-specific questionnaires via e-mail for purposes of collecting sociodemographic and clinical data such as age, sex, working experience and environment. The patient questionnaire included variables on sociodemographic and health-related data. Informal caregivers were also asked about the support they provided to their relative in daily medical care.

### Data analysis

All transcripts were imported into MAXQDA 2018 [53]. Between January and March 2021, interviews were independently analyzed by four researchers (M-SB, TSD, A-AK, LR) using content analysis. This research method is defined as providing subjective interpretations of text data content by systematic classification of coding and identifying themes [54]. The four researchers worked together to code the first two interviews, one from the inpatient and one from the outpatient setting (one IP and one GP interview) and agreed on the main coding tree categories. The main categories were analyzed with deductive coding and new subcategories based on the data itself were created with inductive coding [54, 55]. The remaining interviews were coded by pairs of researchers: TSD and A-AK analyzed interviews from the outpatient care setting, patients and informal caregivers, while M-SB and LR analyzed interviews from the inpatient care setting. The interviews with clinical information scientists and patients/caregivers received their own coding trees, with the main categories similar to those from the inpatient and outpatient settings. Sub-categories added by inductive coding were interpreted by consensus of the above-mentioned researchers.

### Researcher characteristics

Researchers’ assumptions and interests may have influenced the analysis and interpretation. The authors’ characteristics are: TSD (female): health scientist and researcher; MH (female): physiotherapist, health scientist and researcher; A-AK (female): psychologist and researcher; M-SB (female): physiotherapist, health scientist and researcher; LR (female): pharmacist and researcher; JP (female): physiotherapist, study assistant; FB (female): nurse, study assistant; SSR (female): study assistant; SK (female): pharmacist and researcher; CM (female): MD, public health specialist, researcher, professor of general practice; HS (female): pharmacist, researcher and professor (clinical pharmacology and clinical pharmacy); JE (female): health scientist and researcher; KM (female): health scientist and researcher; KV (female): health scientist, sociologist and researcher; MvdA (female): health scientist and epidemiologist, researcher, and professor of polypharmacy and health services research.

## Results

### Sample

Table 1 defines the nine stakeholder groups and lists the characteristics of the 32 participants who completed semi-structured expert interviews and questionnaires, including 20 (63%) women. Twenty-eight of the participants were HCPs and four were patients or informal caregivers. HCPs had a mean age of 42 (range 30–62) years and reported average work experience of 15 (range 1–40) years. The mean age of the patients and CGs was 68 (range 48–79) years. The average interview duration, excluding welcome, introduction, technical notes and data privacy comment, was 31 minutes (range 12–46 min.). Due to technical problems, one interview was interrupted and resumed after a few minutes. HPs in our study are pharmacists who are hospital employees assigned to specific wards, who participate in ward rounds, perform medication reconciliation, and/or deliver medications to the wards [56, 57]. Although this type of HP is not yet common practice in Germany, they serve as role models for good practice in interprofessional inpatient working settings. Compared to other countries [58], for example, HPs in this study do not have the authority to prescribe medication, and physicians also conduct the discharge interview with patients. CISs in our study were employed at hospitals and had different professions such as medical information scientist, internist or chief medical information officer.

**Table 1:** Participant characteristics (n = 32).

Care setting	Sex (female), n (%)	Age (years)		Work experience (years)	
		Mean±SD	Range	Mean±SD	Range
**Inpatient care setting:** Inpatient physicians (n = 4), inpatient nurses (n = 4), hospital pharmacists (n = 3), clinical information scientists (n = 3)	6 (19)	40.07±7.74	30–62	12.64±10.36	2–40
**Outpatient care setting:** General practitioners (n = 6), healthcare assistants (n = 4), ambulatory care nurses (n = 4)	10 (31)	45.14±9.15	32–60	16.50±7.74	1–27
**Patients (n = 3) and informal caregiver (n = 1)**	4 (13)	67.50±11.93	48–79	n/a	n/a

### Expert interviews

**Fig 1:**
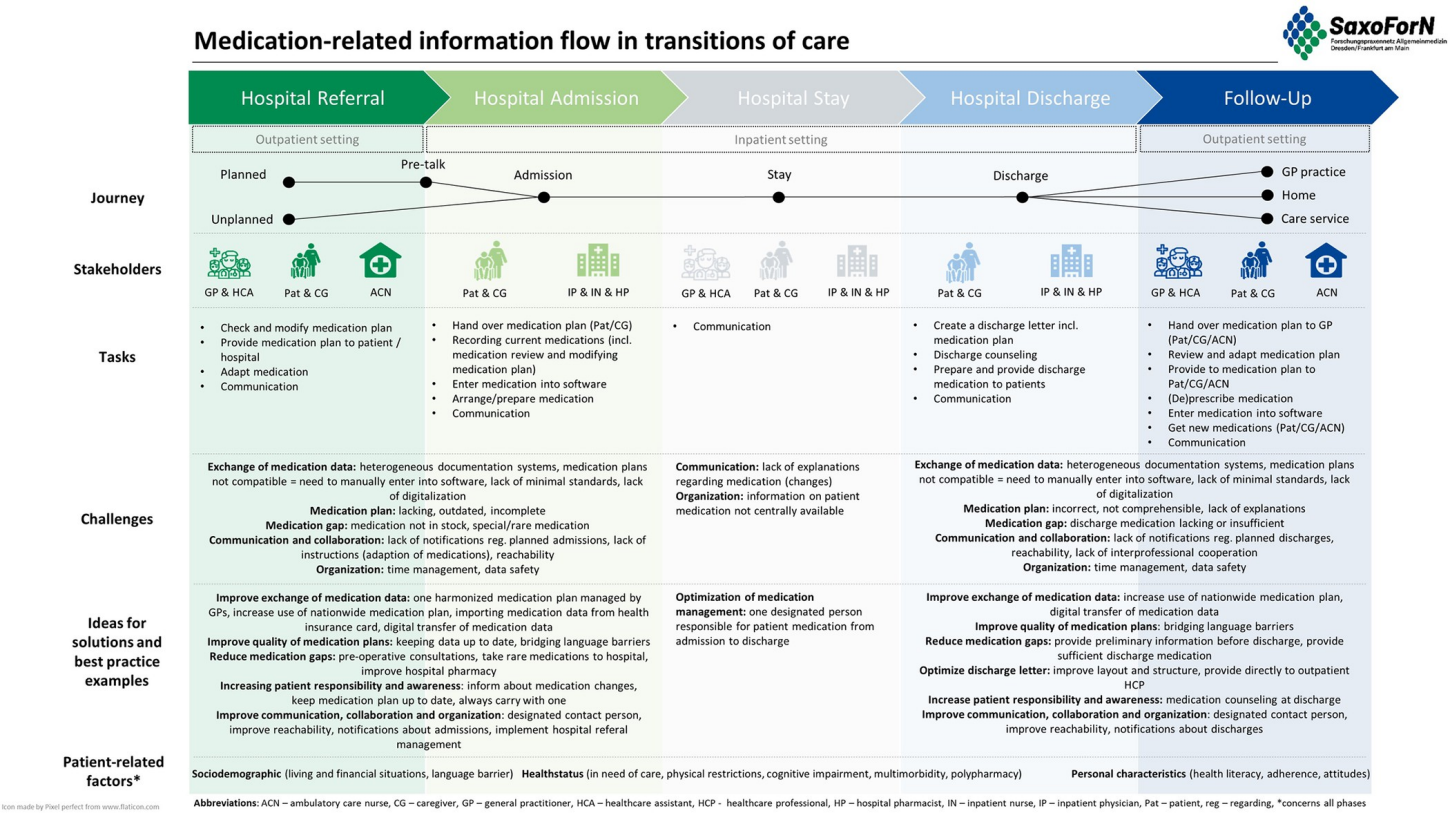
Patient journey map.

The topics of the expert interviews represent the main categories of the coding tree. The findings of the analyses are summarized in a patient journey map (Fig 1). During the interviews, participants also reported on issues generally associated with transitions of care. In accordance with our research objective, we only present the results concerning medication management in this paper.

#### Topic 1: Stakeholders and tasks

Participants identified key stakeholders involved in transitions of care. In the following, we describe medication-related tasks of stakeholders during transitions of care. These might differ depending on the patients’ individual journey and whether the hospital admission was planned or unplanned.

*Hospital referral*. GPs reported that for planned hospitalizations they check the medication plan for currentness and completeness, and modify it as needed:

*“Is [the medication plan] up to date? […] Has the dosage changed? Occasionally some specialist findings have been added to it. […] And whether something else needs to be included for self-medication.” (GP06f)*

The updated medication plan must be printed and handed over to patients who are supposed to take it to the hospital. If needed, the GP may adapt medication prior to hospital admission (e.g., anticoagulants). Sometimes, GP practices try to provide information to the hospital staff in advance, for example, in complex cases. Most GPs stated they were responsible for the medication plan; some responded that they were supported by their HCAs. Other indicated tasks of HCAs included answering questions from patients or forwarding questions to the GP.

GPs sometimes directly refer patients from the GP practice to the hospital (unplanned admissions). In other cases, they may be informed by an ACN or caregiver about a patient’s health deterioration and refer patients without seeing them, for example, in the case of emergencies. In these cases, relevant documents are handed over to the emergency physician, directly sent to the hospital by GPs or taken to the hospital by caregivers.

*“If in an acute situation, I refer the patient to the hospital via ambulance right from my office, they don’t have everything with them or whatever. In that case I then usually have to inform third parties as well who, for instance, also have to help out.” (GP05f)*

Ambulatory care nurses stated that their tasks include preparing an up-to-date medication plan which they retrieve either from their own software, or if not input in their system, from the patient or from the GP practice. If patients have medication plans from different specialists, all plans are copied and handed over to the hospital staff or the emergency physician. Prior to planned admissions, ambulatory care nurses contact the hospital to ask about medication that needs to be provided for the first day.

*Hospital admission*. When the patient enters the hospital, one of the main stakeholder groups involved is that of inpatient physicians, who are responsible for checking the medication plan and adapting the medication if needed. They also enter the medication into the in-house software and arrange its distribution. Inpatient nurses support inpatient physicians by both recording patients’ current medications and by communicating with stakeholders from the outpatient care setting. In addition, INs are responsible for ordering medication.

*“If there are any medications lacking, there are other wards, so it’s normally pretty common to just go somewhere else to borrow some, at least for one dosage.” (IN04m)*

Hospital pharmacists saw their responsibilities in recording patients’ current medications (including communication with the patient, GPs, ACNs and CGs), performing medication reviews and entering medication data into the in-house software system. They consult inpatient physicians and nurses on medication-related questions or in case of inconsistencies.

*“[…] if there are any interactions, anything that [the inpatient physicians] need to be aware of; yeah, some of them come to us of their own accord too and just want us to discuss some things with the GP again […]–why the patient is now taking something, for example, that cannot be deduced from the diagnoses, right off, so to speak.” (HP03f)*

Patients and caregiver in our study described their responsibilities in taking their medication and medication plan to the hospital and handing them over to the professionals.

*Hospital discharge*. For the transition back to the outpatient care setting, a discharge letter including an updated medication plan is created. Inpatient physicians described that this includes reconciling the medication plan from admission with the one saved in the in-house software, and creating a new plan with notes and explanations for the GP. They also contact the GP practice in special cases (before weekends, during holiday season and/or in case of expensive or special medication) to ensure continuous medical care of the patient after discharge. Lastly, their tasks include counseling the patient at discharge:

*“Ultimately, a counseling session is held with each patient at discharge, and they are also given the medication plan with the discharge documents, where the new or changed medications are explicitly addressed and an explanation is provided to the patient on why this is now the case.” (IP03f)*

Inpatient nurses reported that their tasks include providing the discharge medications according to the new medication plan and checking them once delivered by the hospital pharmacy. INs reported that narcotics are not provided by the hospital pharmacy and are therefore manually added to the discharge medication. INs also support inpatient physicians in discharge counseling (explaining and handing over the new medication (plan)). Moreover, one IN reported that in this capacity nurses also critically review the medication plans and address IPs if anything needs clarification.

*“Where the inpatient nurses are also increasingly watching out is with all the pills–do they really have to continue being taken in the same way? Or can one maybe even reduce certain things in advance?” (IN01m)*

Hospital pharmacists indicated that they review the discharge medication, prepare the medication plans including any notes for the GP (e.g., on changes, dosage, interactions and/or monitoring) and check the route of administration.

Sometimes inpatient physicians and hospital pharmacists discuss continuation and deprescription of medication before finalizing the discharge medication plan. HPs stated that they are sometimes involved in the discharge talk with the patient or in the organization of patients’ medical care after discharge together with any number of other stakeholders from both the inpatient and outpatient care settings.

*Follow-up*. The patients and the one informal caregiver participating considered it their responsibility, after the patient has been transferred back to outpatient care, to hand over the medication plan to the GP at the follow-up appointment (usually one day after discharge) and to collect new medication at the pharmacy. Among other tasks, GPs critically review the new medication plan and adapt it if needed (deprescribing medication, correcting dosages, changing the order, accepting or rejecting new medication).

*“[I] think about the changes and take them on board or don’t even adopt them if I don’t think they make any sense.” (GP04m)*

Further GP tasks include entering medication changes into their practice’s software system, handing over the new plan to patients, and if applicable, prescribing and monitoring new medication. GPs stated that all medication changes generate further tasks because they include communication with other stakeholders, e.g., with patients (explaining changes, demonstrating application/use of new medication (devices), such as inhalers, estimating whether the patient is able to implement new changes and if not, arranging support), caregivers and ambulatory care nurses (informing them about changes and giving medication-related instructions), community pharmacies (ordering medication if patient is unable to personally collect it and asking the pharmacies to hand such medications over to patients or ambulatory care services) and inpatient physicians (asking questions related to the medication plan or changes).

HCAs noted that they support GPs with both the new medication (plan) (e.g., comparing old and new medication plans, entering medication changes into the practice’s software system), and in communication with the patient (e.g., explaining and demonstrating new medication) and with inpatient nurses (e.g., forwarding questions from the GP).

Ambulatory care services forward the medication plan to the GP practice and organize new prescriptions and the medication itself.

*“The GP is asked to prescribe the medications that are not in stock. In the best case, the prescriptions are collected immediately by us. If the GP is far away from us, they send […]the prescriptions by letter to the patient.” (ACN04f)*

#### Topic 2: Challenges

Participants identified numerous medication-related challenges described in the following.

*Exchange of medication data*. Participants criticized the lack of standardized structure and unified digital solutions for the exchange of medication data, which means that data often has to be transferred manually and medication plans are partially or completely handwritten. This is very time-consuming and also prone to errors, especially under time pressure.

*“[…] digital file may be clearer because it is centrally stored, but it also requires a lot of clicking around when it comes to data maintenance and input […], so that that soon outweighs the advantages. […] Transferring this information into the system–whether it’s a paper, a file or a digital file–is a huge time factor and also often requires a high level of concentration, which can very quickly result in typing errors.” (IP01m)*

*“We then have to enter the medications manually in our software system. […] This method is pretty error-prone. […] If there is an error in the medication record or medication plan or we make a mistake in entering it in our documentation, then the error gets transmitted all the way to hospitalization.” (ACN01m)*

As a result, patients often act as conduits of information from one care setting to another, which was viewed by both inpatient and outpatient stakeholders.

One clinical information scientist remarked that the exchange of information is insufficiently digitalized and that HCPs mostly rely on paper-based communication tools such as fax and e-mail.

One healthcare assistant highlighted concerns regarding the seamless transfer of medication plans:

*“That is another ‘black box’ thing. Of course, medication plans are printed out in the hospital for the patient to take with them and give to the GP. But whether such a medication plan actually makes it to the GP is always a question anyway.”(HCA01f)*

*Medication plan*. Currently, paper-based medication plans are used for exchanging information on patients’ medication between inpatient and outpatient care. However, participants noted a variety of problems relating to these plans. For example, medication plans may be outdated or incomplete (e.g., because medication prescribed by other HCPs or over-the-counter medication is lacking).

*“In the case of patients who come in, where the medication plan may not have the most current date […], where you then simply approach them, for instance, and ask: ‘Is this still the current dosage?’” (HP03f)*

Other problems referred to lack of medication plans, especially in the case of unplanned admissions. This is additionally challenging when patients are unable to provide valid information about their own medications and hospital staff does not have any contact person from the ambulatory care setting.

*“Then often they don’t have any lists directly with them and they are also unable to directly tell me what they have taken lately.” (IP02f)*

*“And if, say, it’s maybe around 1:00 p.m. on Friday, then it may be extremely difficult to get this information under certain circumstances, because the GP can usually no longer be reached.” (HP02m)*

GPs, in turn, remarked that medication plans after discharge are not always comprehensible, e.g., due to a lack of explanation of changes or new medications. In this context, inpatient stakeholders explained that medication plans may sometimes be incorrect or not up to date at discharge because of short-term medication changes or patients being discharged on short notice.

*“In itself, I think, there is just still such a problem, where we also then don’t keep up with the situation that the inpatient physicians also like to just completely change something again at discharge or reconsider that they want to give half a tablet more after all.” (HP03f)*

*Medication gap*. Inpatient stakeholders reported that medication gaps occur at hospital admission, for example, due to a smaller variety of drugs listed on the hospital formulary; hence some are not readily available as not in stock. This restriction requests substitutions or (time-delayed) procurement via a wholesaler. Concerns related to medication gaps were also noted as arising at hospital discharge:

*“It gets difficult when medications themselves are not available in the hospital, that you cannot give enough of, which is often the case right before weekends. […] or in the case of certain drugs that you are not allowed to give patients to take with them, like morphine patches containing opiates.” (IN01m)*

Outpatient stakeholders reported that the amount of discharge medication varies greatly and that it is challenging for patients as well as for ambulatory care services to organize medication in time.

*“Often [patients] come on Friday afternoon and you don’t organize anything then. That means you have a gap in medication administration […] In the worst case, this can be painkillers or antibiotics.” (ACN03f)*

*Communication and collaboration*. Participants perceived communication and cooperation with others as challenging, both within and across care settings–there is a lack of notifications to all involved stakeholders, e.g., regarding planned admissions or planned discharges. Especially ACNs stated they are not well informed, which results in organizational and time-related problems. Another deficit concerned specific instructions, e.g., related to medication adaptations before admission:

*“Coordination with the physicians, especially about anticoagulation. […] It [is] still so that you have to make phone calls afterwards. Of course, this is futile and also time-consuming.” (GP01f)*

Phone calls are often necessary, but it is difficult to reach the person in question and get the desired information, especially in emergencies, on evenings and weekends, resulting in information gaps that can only be filled with a delay.

*“I would say that communication doesn’t work very well. […] You are then always put on hold […] or they have phone hours at times when I might also make house calls sometimes.”(GP05f)*

One ACN added:

*“While we do have an emergency phone, of course we don’t actually go back to the office at 8:00 p.m. to fax something. […] That’s always a bit difficult with unplanned cases.” (ACN01m)*

Furthermore, participants criticized the lack of interprofessional cooperation. One ACN noted that IPs prescribe medication without *“paying attention” (ACN03f)*, while GPs and HCAs complained about double prescriptions or prescriptions for medication that is not permitted to be prescribed by outpatient physicians.

*“That they sometimes prescribe things that the patient has already had before, that is to say that something gets prescribed twice.” (HCA02f)*

*“Then […] occasionally medications also get prescribed that have no approvals for the [outpatient] setting.” (GP04m)*

GPs also reported experiences in which inpatient physicians often make adaptations that the GPs perceive as unnecessary (e.g., interrupting anticoagulation) or even harmful to patients:

*“[…] unfortunately there are relatively frequent problems at the interface, yes. […] Often they go to the hospital with 10 and come back with 15 [medications].” (GP04m)*

Inpatient stakeholders, in turn, had the impression that their suggestions on medications are not welcomed by GPs:

*“Most GPs just do their thing then. […] They look at their budget and think “Oh, that medication is too expensive for me”, and simply discontinue it.” (IN04m)*

From the perspective of patients, there is a lack of communication regarding medications during the hospital stay. One patient would have liked to have had an explanation of her medications:

*“These are your medications.’ But another explication then of who and what–[…] there wasn’t any […] I asked the night-shift nurse: “What am I actually getting?” […] and the night-shift nurse said to me: ‘You don’t need to know that; you can’t remember it anyway.” (Pat02f)*

*Organization*. Organizational problems referred to time management, internal procedures and data safety. For example, ACNs and HCAs reported that time constraints are challenging for making arrangements or preparing documents before admission, as well as for assuming care (ACNs) or for providing timely appointments after hospital discharge (HCAs).

*“This is sometimes difficult for us, because we don’t always immediately have an appointment free for the patient on the day of discharge or the following day.” (HCA04f)*

Insufficient internal processes reported by inpatient stakeholders included, for example, the observation that changes in medication at admission are not transparently documented or that medication data is not centrally available for all inpatient stakeholders.

*“We often have the problem that you first have to collect your information […], that all information is […] available somewhere on the ward but perhaps not yet at the moment when I need it, at least not centrally.” (HP03f)*

In case of unplanned admissions, one hospital pharmacist also reported that medication plans get lost in the emergency room:

*“The minor problem we still have now is, especially when the patients come in via the emergency room. The medication plans are not scanned there and get lost.”(HP01f)*

Concerning data safety, HCAs felt that it is difficult to maintain confidentiality in telephone calls with hospital staff while other patients wait at the counter in the GP practice. One GP, moreover, felt that data safety hinders him from receiving necessary information:

*“This data safety, yes, that just interferes, it is a hindrance and that is then sometimes very difficult. Because it’s hard to get any information.” (GP06f)*

#### Topic 3: Solution ideas and best practice examples

The stakeholders mentioned a variety of ideas for solutions including best practice examples for overcoming medication-related challenges.

*Solutions for improving the exchange and sharing of medication-related information*. Various stakeholders emphasized that medication-related information should be more easily shared, preferably through digital solutions. While one IP proposed the idea of having one harmonized medication plan “*[…] if possible something like a single sheet*, *so that the patient doesn’t have five forms*.*” (IP03f)*, other stakeholders were already aware of the nationwide standardized patient medication plan and suggested increasing its use in the inpatient setting and at discharge. In particular, they saw great advantages in the QR code, which can be scanned when patients are admitted to hospital and helps in transferring the medication data into the in-house data software system:

*“If every patient came in with a nationwide standardized medication plan that was scanned once on admission and was then in the… [system], then it would be great–that would be the optimal solution for us.” (HP03f)*

One HP suggested that the medication plan should be managed by GPs but open to other physicians to add medications. In general, digital solutions were preferred in order to minimize the errors associated with entering medication data and to directly transfer patient data between different software systems. There was also the wish to import the medication plan from the patient’s health insurance card:

*“That is, if such information is stored on a chip card, where everyone has access to it–from the family doctor to the hospital–accessible to all stakeholders. Because then I think no information gets lost.” (IN01m)*

One IP could also imagine to *“record the medications verbally*, *which are then automatically and digitally transcribed into the in-house software system*.*” (IP01m)*

*Optimizing medication management within the inpatient setting*. One IP desired that a designated person within the inpatient setting be responsible for medication management from admission to discharge:

*“[…] that would have to be a kind of liaison, one not involved in the normal daily process, […], but specifically responsible for it. And that also knows a little bit about the medications and that one organizes in such a way that the medications, which are then valid until the day of discharge, that they are then also integrated into a proper plan and perhaps given again separately to the patient.” (IP02f)*

*Improving the quality of medication plans*. Stakeholders from both inpatient and outpatient settings (IP, IN, GP, HCA) identified potential for improving the quality of medication plans:

*“[…] We as HCAs should always try to reconcile the plan or with the patient every time. When the patient picks up a prescription always asking how the medication is: ‘Are you taking it exactly as we have it specified in the plan?’” (HCA02f)*

One HCA proposed the idea of bridging language barriers by compiling a list of translations and explanations of medications in different languages.

One IP and one IN shared a *best practice example* for dealing with ambiguous medication plans at admission, i.e., it is better to leave it out than to give the wrong medication:

*“In case of doubt, if it seems strange to me and the patient doesn’t really need it because all their vitals are ok for the time being, I prefer to leave it out than administer the wrong thing, that is, until I’ve found a more reliable source.” (IP04f)*

*Reducing medication gaps*. One HP mentioned that *“pre-operative consultations” (HP02m)* for elective surgeries can help to avoid medication gaps that occur due to rare and uncommon medications.

One IN suggested that patients should *“bring such rare and uncommon medications” (IN02f)* to the hospital at admission.

Another IN further suggested that hospital pharmacies should improve availability.

*“Either the pharmacy is really staffed 24 hours a day […] or there is maybe even one person on duty who can briefly activate the unit-dose dispenser.” (IN04m)*

In order to avoid medication gaps at discharge, one GP and one ACN suggested informing outpatient setting stakeholders early of a patient’s discharge date and providing a preliminary medication plan to ensure that HCPs have enough time to prepare and have everything they need in stock.

*“Well, it would be very nice to receive the discharge letter as early as two days in advance, or at least a preliminary version, or the medications.” (GP03m)*

Patients, HCAs and ACNs proposed providing enough medications or a prescription at discharge so that an immediate consultation at the GP practice–particularly difficult before weekends or during holiday season–is not required.

*“My experience was positive because there was a weekend in between and I got [the medications] for three days. Otherwise you only get them for one day.” (patient02f)*

One GP proposed reducing medication-related knowledge gaps in inpatient HCPs by providing more information on prescription medication.

*“That the hospitals would also know what is possible and what is not within the scope of the mandatory health insurance scheme. […], because regulations on medications or prescriptions in the outpatient setting have become even stricter. You can’t prescribe omeprazole or pantoprazole to just anyone.” (GP04m)*

One best practice example was shared by a patient looked after by a welfare institution which supported her in organizing medications after her discharge:

*“Then they had them there–the C-organization is now taking care of me, and there they arranged to obtain the medicines, so that I had no problems there that any would run out in the meantime.” (patient01f)*

*Optimizing the layout and distribution of the discharge letter*. Stakeholders from the inpatient and outpatient setting suggested several ideas for improving the discharge letter, emphasizing organized layouts based on *“diagnoses and indications”(IP02f)*.

Moreover, ACNs and IPs suggested sending a duplicate of the discharge letter including the medication plan directly to outpatient HCPs.

*“One hospital, for example, does it in such a way that a copy of the letter goes to us. We are not dependent on the GP’s discharge letter […], but have a letter ourselves that we can put in the patient’s file and where we have the current medication plan stored with us.” (ACN04f)*

In addition, GPs, HCAs and ACNs expressed a desire for final discharge letters at the time of patients’ discharge from hospital.

*“If we just get the letters in a timely manner–the completed ones that is, not just the preliminary letters. If it is then also about other things, which perhaps are still yet to follow, what one should do, so should arrange, or further examinations.” (GP05f)*

*Increasing patient responsibility and awareness*. One HCA expressed a desire to see an increase in patient responsibility and awareness, e.g., informing HCPs about medication changes and keeping their patient medication plans up to date, e.g., regularly obtaining an updated medication plan from their GP and carrying it with them at all times in preparation for an unplanned admission.

*“[…] cooperation on the part of the patient. That they also tell us when they no longer take a tablet, that they then say: ‘Here, I didn’t take these.’ Or that the patient when they were seen by a specialist, if there is a change in medication, which also happens a lot, that you just get notified by the patient […].” (HCA02f)*

One patient would also have preferred to be more involved and proposed a *“discharge medication conversation” (patient01f)* to ensure that patients are informed of any medication changes during their hospital stay, including explanations regarding indications and (inter)actions. Another patient also wished for an *“opportunity to read their discharge letter” (patient03f)* before discharge and would have liked an opportunity to discuss medication changes with the physician.

#### Improving communication, collaboration and organization

Stakeholders from both the inpatient and outpatient setting identified the need to improve communication and collaboration across care settings and considered it important for the continuity of care. GPs and Ips desired a designated contact person and improved reachability.

*“[…] good, when a patient is admitted to the hospital, to send the GP a notification as well–standardized. […] because every GP would like that.” (GP03m)*

The need for more direct communication between the inpatient setting and GPs was confirmed by one ACN. In addition, ACNs wanted to be involved in the process and informed of planned admissions.

*“It is difficult that the wards try to go through us sometimes, which would actually be better if they would clarify this with the GP practices.” (ACN02f)*

One idea for improving the organization of admissions included implementing a *“hospital referral and admission management” system (ACN03w)* to ensure relevant patient information on personal items such as clothing.

#### Topic 4: Patient-related factors

Stakeholders shared their views on general patient-related factors that hinder or facilitate the medication-related process in transitions of care.

*Sociodemographic*. Stakeholders mentioned that patients’ living and financial situations play an important role in this context. Language barriers also make effective communication difficult, and the limited availability of interpreters further complicates the situation.

*“In the case of people with family members who care, you get good information then too and can also work well together.” (IP03f)*

*Health status*. Stakeholders emphasized the vulnerability of patients in need of care and pointed out that they are dependent on caregivers or family members to administer medication. In addition, cognitive impairment poses a challenge to reliable information.

*“In some cases, patients no longer take care of this themselves, but are given the [medications] by the nursing service caregivers or relatives, for instance, and then just take them. Oftentimes, they don’t even know what medications [they] are taking.” (IP02f)*

One inpatient physician assumed that a large number of conditions and medications for a single patient has a negative impact on the validity of information.

*“Of course, the number of medications is also a crucial issue. Someone who takes only one medication can of course provide more information about it than say these poly-medicated and multimorbid patients who come with like 20 drugs. The more drugs a patient has, the more likely I think the information we get is prone to error.” (IP01m)*

In the case of unplanned admissions, it is especially challenging when patients are unable to provide valid information on their own medications due to their medical condition, especially when they are unable to consent to disclosure of their health data.

*“Another complicating factor is that the external institutions still [require] a signed […] declaration of consent from the patient for transfer of the data. But if the patient is so ill at the moment, they may not be able to sign it at all. Then I need the caregiver again, but I can’t reach that person at night.” (IP04f)*

*Personal characteristics*. One of the main influencing factors with regard to patients referred to their willingness to be informed, to manage their medications, to be actively involved and to adhere to therapy plans.

*“The educated, well-sorted out, organized and responsible patient. So, an adult who knows what things they take and at what time and in what dosage.” (IP04f)*

Stakeholders often found it difficult to tell whether information provided by patients is reliable:

*“Otherwise, of course, when you talk to patients, you always have to rely on what the patient wants to tell you. Yes, so you never have a one hundred percent guarantee that the patient will tell you everything.” (HP02m)*

Patient attitudes, such as insistence on specific medications, can further complicate communication and decision-making.

*“Sometimes, for instance, discussions with the patient complicate things, because they naturally also insist on certain medications. […] (IN04m)*

## Discussion

The findings confirm that the continuity of medication management is endangered by numerous factors which include the non-availability or poor quality of patients’ medication and health-related data, a partial lack of suitable options and legal standards for communication and collaboration between stakeholders within and across care settings, and insufficient use and support of digital solutions. It is worth noting that these views were shared by both inpatient and outpatient stakeholders. The interviews with patients and the one caregiver had a stronger focus on their experiences related to the patients’ most recent hospital stay. A large discrepancy was revealed in ideal and actual transitions of care.

Similar to previous research [59, 60], our interviews also indicate that the information situation at transitions of care is poor. First, this refers to the **quality of information available at both referral and discharge**

In line with Sørensen et al. [59], the information on patients’ medication was found to be insufficient. This refers, for instance, to non-updated medication data, lacking information on medication changes and indications, and even completely missing data. The results further confirm that the quality of information varies greatly from HCP to HCP [59]. Most HCPs used medication plans that were not compatible between software systems of inpatient and outpatient sectors, and that were heterogeneous in terms of content. This often resulted in additional work with an increased susceptibility to error because patients’ medication data needed to be manually transferred into in-house software systems. Although participants noted the need for a uniform medication plan, the nationwide standardized patient medication plan already introduced in Germany in 2016 [61], was rarely used by our participants. However, Amelung et al. [62] revealed that even the nationwide standardized medication plans are incorrect in 78% of admitted patient cases. In line with previous research [26, 63], we point out the significant role of family members in managing medication for older patients during transitions of care. Manias et al. [63] indicated that family members were not always adequately included by HCPs in the information exchange and that there is a tendency to overlook the medication-related efforts made by families. Communication regarding medication plans and their implementation during transitions is often disorganized or lacks shared decision-making between families and HCPs [63]. Furthermore, to enhance the seamless transfer of information and improve information quality, the WHO [15] emphasizes the importance of having adequate resources, such as HCP staff, including clinical pharmacy staff. In addition to that, education, training, and the availability of digital tools for both patients and HCPs are facilitating factors. Such resources and tools are sorely needed to counteract limited workforce capacity and skills, which often constitute a barrier to enhancing medication safety at transitions of care [15].

Based on the limiting factors presented above, our study emphasizes the **urgent need for implementing legal standards for and a convenient manner of expediting the exchange of medication and other health-related patient data within and between HCPs from different care settings**. This particularly refers to the medication plan currently described as incompatible between and within care settings. Similar to Tolley et al. [60], we too found that stakeholders maintain data in their own in-house systems, with the flow of information between different systems unidirectional and in some cases not even feasible. As reported in that study, HCPs in our study were concerned about this fact as well, particularly with regard to the lack of information in unplanned admissions. Denmark, for example, has a “Shared Medication Record” that displays the current medication of a patient in real time, visible for HCPs from both inpatient and outpatient care settings. While GPs there are obliged to update patients’ medication data before hospital referral, inpatient physicians are required to electronically provide the discharge letter to the GP within one to two days after discharge [59]. In our study, however, HCPs from both inpatient and outpatient care settings reported that they rely on patients carrying the information provided by an HCP from the other care setting. The majority of participants, including patients and the one caregiver, considered this procedure unreliable and inappropriate. In this context, our participants wished there were an option for messages and information to be directly sent to other HCPs between software systems.

Furthermore, our study revealed the lack of **suitable means of communication and collaboration between stakeholders, including patients**. This often results in duplication of/overlapping work, as the study revealed that multiple HCPs assumed some of the same tasks. Participants also criticized a lack of options for directly contacting the HCPs involved, and delayed or even lacking notifications about patients’ admission to and discharge from hospital. Concurring with the results from Sørensen et al. [59], particularly ambulatory care nurses feel insufficiently informed and left behind. A British study also found the need for better communication [60]. In general, our study participants from the inpatient setting suggested a stronger involvement of hospital pharmacists. In this regard, results from a Danish study [59] indicated that including hospital pharmacists in transitions of care can help in identifying and reducing medication-related problems and medication errors after discharge. Similar to findings from a Slovenian study [64], our results show that collaboration with pharmacists frequently occurs in the inpatient setting. In the outpatient setting, for example, medication reviews by pharmacists and/or in collaboration with GPs can reduce the total number of medications, including potentially inappropriate medications and potential drug-drug interactions [65, 66]. Overall, existing research [25, 67] indicates that hospital pharmacists, community pharmacists and pharmacy technicians, who are highly qualified to prevent and manage medication-related problems while ensuring the safe and effective use of medications, are not sufficiently involved in many Central and Eastern European countries. It is worthy of note at this point that the Czech Republic and Slovenia, for example, are leading the way in the successful implementation of clinical pharmacy services [67]. In Germany, task-sharing between GPs and community pharmacists for management of medications in the outpatient setting has already been tested in pilot projects [68]. Finally, similar to previous findings [59], patients in our study felt that they were not adequately involved during their hospital stay and particularly criticized the lack of medication talks at discharge.

Overall, all study participants emphasized the need for digital solutions for increasing continuity in medication management in transitions of care. This requires creating digital interfaces to better support interprofessional collaboration and the exchange and sharing of patient data between different systems and care settings. Furthermore, data should be shared in real time and in a standardized, uniform manner. It should be noted that the proposed changes are generally covered in the current German plans for the implementation of a telematics infrastructure aimed at networking all players of the healthcare sectors and enabling communication among them [69]. It nevertheless remains essential to investigate whether these possibilities will be fully exploited in routine care.

This study has several strengths and limitations. In terms of strengths, one key strength is the inclusion of a large and heterogeneous set of nine different stakeholder groups from the inpatient and outpatient settings, including patients and one caregiver. This allowed us to capture a wide range of experiences and perspectives on medication management in transitions of care. Moreover, involving key stakeholders at this stage enabled us to prepare the co-design of the planned intervention that takes the stakeholders’ specific circumstances and needs into account. This could support feasibility, acceptance and implementation of the intervention.

In terms of limitations, as our study focused solely on GP-initiated hospital admissions, we may have missed other relevant aspects with regard to the continuity of medication management that would come into play if we had also included hospital admissions initiated by other physicians. Furthermore, we did not include the perspective of ambulatory pharmacists. We also acknowledge that some of the results, for example, regarding the insufficient use and support of digital solutions, may be very specific to the hospital, department or practice represented by the participants, thus limiting these results’ generalizability to the overall German healthcare system. Another limitation is that of homogeneity and lack of variance in the patients’ and informal caregiver statements. The patient stakeholder group, including one informal caregiver is smaller than the HCP stakeholder groups. However, in the second part of the study, additional participants from the patient group were involved in the planned workshops, making a valuable contribution to the further development of the intervention. This study nonetheless achieved a balance in respondents from the nine different stakeholder groups (3–4 per stakeholder group).

In conclusion, we have obtained comprehensive results demonstrating the potential effects of various facets of medication management during transitions of care. Despite the numerous barriers identified, the present findings have revealed that involved HCPs from both the inpatient and outpatient care settings have a common understanding. Our results were predestined for use in the HYPERION-TransCare study’s sub-study 1, second stage, in which an intervention to improve continuity of medication management was co-designed with stakeholders (HCPs, patients and one informal caregiver) in interprofessional workshops and further tested in a subsequent pilot study (HYPERION-TransCare sub-study 2).

## Supporting information

S1 Checklist Consolidated criteria for reporting qualitative research. (PDF)

S1 File Interview guide GP. (DOCX)

S2 File Interview guide inpatient nurse. (DOCX)

S3 File Interview guide patient. (DOCX)

## Abbreviations

ACN: Ambulatory care nurse
CG: Caregiver
CIS: Clinical information scientist
F: Female
GP: General practitioner
HCA: Healthcare assistant
HCP: Healthcare professional
HP: Hospital pharmacist
HYPERION-TransCare: Acronym of the study “Heading to Continuity of Prescribing in Elderly with Multimorbidity in Transitional Care”
IN: Inpatient nurse
IP: Inpatient physician
M: Male
