# Supplementary material for: Informational continuity of medication management in transitions of care: Qualitative interviews with stakeholders from the HYPERION-TransCare study
Source: PLoS One. 2024 Apr 4;19(4):e0300047. doi: 10.1371/journal.pone.0300047 (PMC10996284; doi:10.1371/journal.pone.0300047)
Supplement: S1 File — (DOCX) [file pone.0300047.s002.docx]

**Interview guide for inpatient nurse**

**Welcome:**

Thank you for your willingness to participate in an interview.

**Questions:**

| **Theme** | **Main question** | **Subquestions** |
| --- | --- | --- |
| **Hospital admission** | As an inpatient nurse, **what** do you have to prepare and organize in terms of medication for a new patient? | *If applicable, ask about any differences between planned and unplanned admissions.* |
|  | **Who** else is involved and to what extent? | *If applicable, ask specifically about communication between those involved. If applicable, ask specifically about the primary care physician.* |
|  | What **tools/checklists/guidelines** are there already to assist you, if any? |  |
|  | Admission medication information: | |
|  | **When** do you receive the information on admission medication? |  |
|  | **How** do you receive the information on admission medication? | *If applicable, ask whether written, verbal, or in what form.* |
|  | **From whom** do you receive information on admission medication? |  |
|  | **What** information do you receive about the admission medication? |  |
|  | Please describe to us **the quality of this information**. |  |
|  | Overall assessment: |  |
|  | What already works **well**? |  |
|  | What **difficulties** do you experience regarding medication in preparing for hospitalization? | How do you deal with them?  *If applicable, have respondents describe the individual situations again in more detail.* |
|  |  | Do you have any **suggestions for improvement**? |
|  |  | What would this require? |
|  | What **patient characteristics** sometimes make it easier or more difficult for you to perform the processes? | *If applicable, have respondents provide patient examples and/or have them describe problems in detail: What exactly are the problems? Can you think of anything else? What were your most recent difficult cases? Which patient groups are more challenging?* |
|  |  | *If applicable, ask for more detail on any factors that facilitate or complicate processes.* |
|  | What **other factors** sometimes make it easier or more difficult for you to perform the processes?  *(personal relationships, a certain environment, certain structures, etc.)* | *If applicable, ask for more detail on any factors that facilitate or complicate processes.* |
| **Hospital stay** | If applicable, for which difficulties regarding medication do you contact the **primary care physician**? | Which information exchange procedure has proven successful in this regard? |
| **Hospital discharge** | As an inpatient nurse, **what** are all the things you have to prepare and organize in terms of medication for a patient’s discharge? |  |
|  | **Who** else is involved on the ward or across wards and to what extent? | *If applicable, ask specifically about communication between those involved.* |
|  | What **tools/checklists/guidelines** are there already to assist you, if any? |  |
|  | What information about their new medications do **patients** receive before they are discharged? | **From whom**? |
|  | What information about **medications** is communicated to primary care physicians? | Are there sometimes queries? If so, what are they? |
|  | To what extent is **continued provision** of medication after discharge ensured? | To what extent do you give patients medication from the ward to take with them? |
|  | Overall assessment: | |
|  | What already works **well** overall? |  |
|  | What **difficulties** do you experience with regard to medication when patients are discharged from the hospital? | How do you deal with them?  *If applicable, have respondents describe the individual situations again in more detail.* |
|  |  | Do you have any **suggestions for improvement**? |
|  |  | What would this require? |
|  | Already addressed at admission:  What **patient characteristics** sometimes make it easier or more difficult for you to perform the processes? | Are there any differences to admission?  *If applicable, have respondents provide patient examples and/or have them describe problems in detail: What exactly are the problems? Can you think of anything else? What were your most recent difficult cases? Which patient groups are more challenging?* |
|  |  | *If applicable, ask for more detail on any factors that facilitate or complicate processes.* |
|  | Already addressed at admission:  What **other factors** sometimes make it easier or more difficult for you to perform the processes? | Are there any differences to admission?  *(personal relationships, a certain environment, certain structures, etc.)* |
|  |  | *If applicable, ask for more detail on any factors that facilitate or complicate processes.* |
| **Final questions** | Can you think of anything else on this topic I may have forgotten? |  |
|  | Is there anything else you would like to add? |  |

**Closing:**

Thank you very much for your willingness to share your thoughts with us. Your responses will be kept confidential and no report based on this data will be associated with you.

Do you have any questions?

Are you interested in further participation in the following workshops?

Thank you very much.
