# Supplementary material for: Informational continuity of medication management in transitions of care: Qualitative interviews with stakeholders from the HYPERION-TransCare study
Source: PLoS One. 2024 Apr 4;19(4):e0300047. doi: 10.1371/journal.pone.0300047 (PMC10996284; doi:10.1371/journal.pone.0300047)
Supplement: S2 File — (DOCX) [file pone.0300047.s003.docx]

**Interview guide for GP**

**Welcome:**

Thank you for your willingness to participate in an interview.

**Questions:**

| **Theme** | **Main question** | **Subquestions** |
| --- | --- | --- |
| **Hospital referral** | As a primary care physician, **what** are all the things regarding medications you need to organize for hospital referral? | *If applicable:* Ask about any differences between planned and unplanned referrals. |
|  | **Who** else is involved and to what extent? | *If applicable:* Ask specifically about communication between those involved. If applicable, ask specifically about the ward staff. |
|  | **What** tools/checklists/guidelines, if any, are there already to assist you? |  |
|  | Overall assessment: | |
|  | What already works **well**? |  |
|  | What **difficulties** do you experience regarding medication in preparing for hospitalization? | How do you deal with them? |
|  |  | Do you have any **suggestions for improvement**? |
|  |  | What would this require? |
|  | What **patient characteristics** sometimes make it easier or more difficult for you to perform the processes? | If applicable, have respondents provide patient examples and/or have them describe problems in detail:  What exactly are the problems? |
|  |  | Can you think of anything else? |
|  |  | What were your most recent difficult cases? |
|  |  | Which patient groups are more challenging? |
|  |  | If applicable, ask for more detail on any factors that facilitate or complicate processes. |
|  | What **other factors** sometimes make it easier or more difficult for you to perform the processes? | E.g., personal relationships, a certain environment, certain structures, etc. |
|  |  | If applicable, ask for more detail on any factors that facilitate or complicate processes. |
| **Hospital stay** | In what medication-related issues are you contacted by the **hospital**, if you are? | Which information exchange procedure has proven successful in this regard? |
| **After hospital discharge** | **What** with regard to medication do you as a primary care physician have to do after a patient’s hospital stay? |  |
|  | **Who** else is involved? | If applicable, ask specifically about communication between those involved. If applicable, ask specifically about the ward staff. |
|  | Are there already any **tools/checklists/guidelines** to assist you after patients have been discharged from the hospital? |  |
|  | Discharge medication information: | |
|  | **When** do you receive the information on discharge medication? |  |
|  | **From whom** do you receive the information? |  |
|  | **What** information do you receive about the discharge medication? |  |
|  | Please describe for us the **means by which you receive discharge medication information**. |  |
|  | Please describe to us **the quality of the information** on discharge medication. | If applicable, ask further questions, such as: How do you deal with this? etc. |
|  | What already works **well** overall? |  |
|  | What **difficulties** do you experience regarding medications after a patient’s discharge from the hospital? | How do you deal with them? |
|  |  | If applicable, have respondents describe the individual situations again in more detail. |
|  |  | Do you have any **suggestions for improvement?** |
|  |  | What would this require? |
|  | Already addressed at admission:  What **patient characteristics** sometimes make it easier or more difficult for you to perform the processes? | Are there any differences to admission? |
|  |  | If applicable, have respondents provide patient examples and/or have them describe problems in detail:  What exactly are the problems? |
|  |  | Can you think of anything else? |
|  |  | What were your most recent difficult cases? |
|  |  | Which patient groups are more challenging? |
|  |  | If applicable, ask for more detail on any factors that facilitate or complicate processes. |
|  | Already addressed at referral:  What **other factors** sometimes make it easier or more difficult for you to perform the processes? | Are there any differences to referral?  (personal relationships, a certain environment, certain structures, etc.) |
|  |  | If applicable, ask for more detail on any factors that facilitate or complicate processes. |
| **Final questions** | Can you think of anything else on this topic I may have forgotten? | Is there anything else you would like to add? |

**Closing:**

Thank you very much for your willingness to share your thoughts with us. Your responses will be kept confidential and no report based on this data will be associated with you.

Do you have any questions?

Are you interested in further participation in the following workshops?

Thank you very much.
