# Supplementary material for: Informational continuity of medication management in transitions of care: Qualitative interviews with stakeholders from the HYPERION-TransCare study
Source: PLoS One. 2024 Apr 4;19(4):e0300047. doi: 10.1371/journal.pone.0300047 (PMC10996284; doi:10.1371/journal.pone.0300047)
Supplement: S3 File — (DOCX) [file pone.0300047.s004.docx]

**Interview guide for patient**

**Welcome:**

Thank you for your willingness to participate in an interview.

**Questions:**

| **Theme** | **Main question** | **Subquestions** |
| --- | --- | --- |
| **Introduction** | You were recently hospitalized ...  How far **in advance was admission to the hospital planned?** |  |
|  | **How long** was your hospital stay? |  |
| **Hospital referral** | **What** were **all the preparations** in terms of medications that you had to make prior to your hospital stay? |  |
|  | **Who**, if anyone, talked to you about your medications or helped you in this regard prior to your hospital stay? | **What help** was offered to you, if any? |
|  |  | Did you have pre-admission interviews or talks **at the hospital** prior to your hospitalization? |
|  |  | Did you have any pre-admission talks with your **primary care physician**? |
|  | **What** was discussed about your medications? |  |
|  | Were you **involved in the talk** in this regard?  How **involved** were you in this regard? |  |
|  | Was this **satisfactory** for you?  How **satisfactory** was this for you? | How did you **deal with** this? |
|  |  | Were there any **unanswered questions** about your medications? |
|  |  | Whom did you talk to about this, if anyone? |
|  | What **type of written information regarding your medications** did you receive for your hospital stay? |  |
|  | Overall assessment: |  |
|  | Thinking back now to the procedures and talks with regard to your medications in preparation for your hospital stay, what would you say went **well**? |  |
|  | Was there anything you **didn’t like so much** with regard to the medications? |  |
|  | How could that be **better** handled in the future? |  |
| **Hospital admission** | Then you were admitted to the hospital.  **Who** talked to you about your medications when you were admitted? |  |
|  | **What** was discussed about your medications? | Were you **involved in the talk** in this regard? |
|  |  | How **involved** were you in this regard? |
|  |  | Was this **satisfactory** for you?  How **satisfactory** was this for you? |
|  |  | How did you **deal with** this? |
|  |  | Were there any **unanswered questions** about your medications? |
|  |  | Whom did you talk to about this, if anyone? |
|  | Overall assessment: | |
|  | Thinking back now to the procedures with regard to your medications at the time of admission for your hospital stay, what would you say went **well**? |  |
|  | Was there anything you **didn’t like so much** with regard to the medications? |  |
|  | How could that be **better** handled in the future? |  |
| **Hospital discharge** | When you were discharged from the hospital, **what** were you told at the hospital about your medications? |  |
|  | Were there any **changes** in your medications? |  |
|  | **Who** talked to you about this matter? |  |
|  | Were you asked for your **opinion** about it? | Were you **involved in the talk** in this regard?  How **involved** were you in this regard? |
|  |  | Was this **satisfactory** for you?  How **satisfactory** was this for you? |
|  |  | How did you **deal with** this? |
|  |  | Were there any **unanswered** questions?  Whom did you talk to about this, if anyone? |
|  | Did the hospital give you any **medications or prescriptions** to take home? | If so, how did this work for you? |
|  | If applicable, did you need any **assistance with medications**? | If so, who provided you assistance? |
|  | What **written information** did you receive about your medications when discharged from the hospital? |  |
|  | How **sufficient** was this information? |  |
|  | How **comprehensible** was the information? |  |
|  | Overall assessment: | |
|  | Thinking back now to the procedures and talks with regard to your medications, what would you say went **well** when you were discharged? |  |
|  | Was there anything you **didn’t like so much** with regard to the medications? |  |
|  | How could that be **better** handled in the future? |  |
| **Follow-up** | Please tell us about your first visit with your primary care physician after hospitalization. |  |
|  | **What** were **all the things you had to do** with regard to your medications after your discharge from the hospital? |  |
|  | **What** did you discuss with your **primary care physician** about your medications? |  |
|  | Were you asked your **opinion** about the medications? | Were you **involved in the talk** in this regard?  How **involved** were you in this regard? |
|  |  | Was this **satisfactory** for you?  How **satisfactory** was this for you? |
|  |  | How did you **deal with** this? |
|  |  | Were there any **unanswered** questions?  Whom did you talk to about this, if anyone? |
|  | Overall assessment: | |
|  | Thinking back now to the procedures with regard to your medications after your hospital stay, what would you say went **well**? |  |
|  | Was there anything you **didn’t like so much** with regard to the medications? |  |
|  | How could that be **better** handled in the future? |  |
| **Final questions** | Can you think of anything else on this topic I may have forgotten? |  |
|  | Is there anything else you would like to add? |  |

**Closing:**

Thank you very much for your willingness to share your thoughts with us. Your responses will be kept confidential and no report based on this data will be associated with you.

Do you have any questions?

Are you interested in further participation in the following workshops?

Thank you very much.
